# Atlanta Academy of Medicine

**Published:** 1873-03

**Authors:** Jas. B. Baird

**Affiliations:** Reporter


### ATLANTA ACADEMY OF MEDICINE.

JAS. B. BAIRD, M.D., REPORTER.

*Extract from Minutes, January* 13, 1873—*Dr. J. P. Logan, President, in the Chair.*

Dr. Taliaferro inquired if there had been any cases of cerebro-spinal meningitis seen recently by any members of the Academy, and if so, requested the histories of the cases.

Dr. Baird responded by saying that he had recently seen a case in which he had an opportunity of making a post mortem examination, and would briefly refer to the prominent symptoms, as requested by Dr. Taliaferro.

A. J. D., white, aged twenty years, intelligent, and until recently lived on a farm. For several weeks had been traveling with a circus company, and was much exposed to cold. When he reached this place, he was taken sick, and came under my observation on November 28. He was attacked about four days before with violent pain in the head, in the back of his neck and spinal column as low as about the sixth dorsal vertebræ, and in ali of the larger joints. There had been no diarrhœa; no epistaxis; no decided fever. There was no tympanitis, though he complained of tenderness when pressure was made over the abdomen. The pain in the head had subsided, but continued unabated in the neck, back, and articulations. Pulse ninety, and good; respiration nominal; bowels constipated; urine highly colored, and very acid; temperature not taken. He could move himself only with great difficulty, and declared the pain much more severe in his ankles, knees, wrists and elbows than in his shoulders or hips. There was no swelling, heat or redness of the joints. Diagnosis, articular rheumatism. Ordered a saline laxative, and one drachm of the bicarbonate of potassa every four hours. About the third day, the potash caused nausea and vomiting, and though it had been continued for forty-eight hours, had produced no alkalinity of the urine. All pain, however, had disappeared, and he was considered convalescent. The next day his pulse was full, strong, and one hundred and twenty per minute. Slight pain in the joints, and slight hyperesthesia of the entire surface, and most severe in the throat, causing difficult deglutition. Ordered a solution containing about twenty grains of bromide of potassium, seven grains of iodide of potassium, and twenty drops of tincture of colchicum three times a day; ten grains Dover's powder as required at night. Following day pulse scarcely perceptible at the wrist; other symptoms unchanged; rested well; complained of soreness of the skin; the irregularity of the pulse as to frequency and force varying from one hundred and sixteen to one hundred and thirty per minute, sometimes good and again imperceptible in the radial artery, together with the hyperesthesia, particularly of the throat, and the rigidity and slight contraction of the cervical and dorsal muscles which now appeared, the existence of cerebro-spinal meningitis was declared. Sinapisms were applied to the spine, and the tendency to asthenia sought to be counteracted by tonic doses of quinine, milk punch and a nutritious diet. Subsequently, a blister was ordered to the scalp and spine, but from the resistance offered by the patient, the result was not satisfactory.

December 10th, 6 o'clock, P. M.—Pulse 128, strong and regular; temperature 102; pupils dilated, respond sluggishly; no pain.

December 11th, 10 A. M.—Pulse 130, good; temperature 101½; pupils unchanged—says he sees dimly, and things look misty; slept well, complains of pain in the head and soreness of the joints; ordered bromide of potassium thirty grains and twenty drops fluid extract ergot three times a day.

December 11th, 6 P. M.—Pulse 120, good; temperature 101; pupils respond readily, seems brighter; slight pain in head; blister reapplied to spine, but again failed to produce vesication; one healthy stool at 3 P. M.

December 12th, 10 A. M.—Pulse very feeble, not able to count pulsations; temperature 99, seems dull and torpid; says he feels easy; rested well; pupils prompt; urine intensely acid, no albumen, no casts, an abundant deposit of the urates, white precipitate with a solution of nitrate of silver.

December 12th, 6 P. M.—Pulse 122; temperature 101: comfortable.

December 13th, 10 A. M.—Pulse 118; temperaturo 100½; resperation 30; rested well after taking twenty grains of dover's powder.

December 13th, 6 P. M.—Pulse 120, irregular; temperature 102; restless.

December 14th, 10 A. M.—Pulse 124; temperatere 100; respiration 36; passed restless night, active delirium reported by nurse. Had twenty grains dover's powder during night; complains of pain in the head and a feeling of prostration, also a very dry mouth; ordered an emulsion containing fifteen drops of oil of turpentine three or four times a day.

December 14th, 6 P. M.—Pulse 120; temperature 99½; quiet.

December 15th, 10 A. M.—No pulse; temperature 100½; rested well without opium. Is now taking bromide of potassium, grs. xxv; fluid extract ergot, gtts. xx; oil turpentine, gtts. xv;—four times a day, and four grains of quinine daily; whisky as required; beef tea, animal broths, milk punch and egg-nogg.

December 16th, 10 A. M.—Pulse 124, feeble, flickering; temperature 100½; rested well; no opium; pain in head and back; severe sinapism to neck.

December 16th, 6 P. M.—Pulse 122; temperature 101.

December 17th, 10 A. M.—Pulse about 90, feeble and irregular; temperature 101; rested well, after taking ten grains dover's powder.

Constipation was relieved easily from time to time by a mild laxative. Nausea existed only for a few hours during the early part of the attack; no ecchymosis, photophobia very slight; slight opisthotonos continued throughout; urine passed involuntarily for the last week of life. Died at 7 P. M., December 24th, having gradually lost strength from the beginning; he steadily sunk until life became extinct. There were no convulsions and no paralysis, though there was from the first a marked weakness of muscular contraction. Intelligence clear to the last. *Sectio-cadaveris eighteen hours after death;* body very much emaciated; meninges of the brain intensely congested; a considerable amount of effusion into the cavity of the dura mater; a thick patch of exudation, resembling a large abscess, covered the inferior surface of the left lobe of the cerebellum, extending, to a limited extent, to the right lobe, and enveloping completely the medulla oblongata, and spinal cord as low down as could be reached with the scalpel, from the cranium into the spinal canal. The pia mater covering the base of the brain presented an unhealthy aspect, being injected, and at some points broken down in texture. The substance of the brain seemed healthy.

Dr. Taliaferro.—Has recently seen a case in which there was evidently some trouble about the brain. The pupils were dilated —insensible to light. There was great difficulty in swallowing, which was not dependent upon inflammation of the fauces, but seemed due to a paralyzed condition of the muscles concerned in the act of deglutition, allowing an accumulation of mucus in the throat. Death ensued in a few days. He inquired if muscular rigidity and the hyperæsthesia of the surface were invariable in meningitis.

Dr. Jno. Stainback Wilson regarded these symptoms pathognomonic of the disease. The cases he had seen terminated within three days.

Dr. A. W. Griggs.—Rigidity, in his experience, is very common, and is a valuable diagnostic symptom, when it occurs in connection with other symptoms. Thinks it is most marked when the base of the brain is the seat of the trouble. It frequently precedes other symptoms. There is not much swelling of the joints, though frequent tenderness. He mistook one case for rheumatism at the outset. An initial chill has generally been observed in cases coming under his observation. The condition of the pupils is variable; now attaches but little importance to their condition. Hyperæsthesia of the throat not very common in his experience, though there is frequent difficulty of deglutition. His cases sleep a good deal. In one case of a child, aged seven or eight years, permanent contraction of the masseter muscles resulted. Two years after the attack a spoon could not be introduced between the teeth. One case, six years of age, was left permanently deaf; several years have elapsed, and now he has almost forgotten how to talk.

Dr. Taliaferro suggested veratrum viride to control the pulse and relieve vascular action.

Dr. J. T. Johnson.—Has seen veratrum in cases of great prostration produce vomiting, yet exert little or no effect on the pulse.

Dr. Taliaferro thought the vomiting must be due to some other cause. He has never seen a case in which veratrum was given to the extent of producing vomiting without exerting a decided influence over the pulse.

Dr. Griggs.—Has frequently used veratrum with satisfactory results after venesection. Can readily understand how veratrum can produce emesis and fail to control the pulse. Has seen it produce vomiting when administered hypodermically after opium poisoning.

Dr. Taliaferro thought that veratrum does not exhaust. The lancet does. Some urge the use of opium in this disease. He can not understand how opium can cut short cerebral congestion.

Dr. Griggs thought if opium was used at all, it should be with the hope of aborting the disease by breaking up the state of innervation by stimulating the vessels of the brain. Quinine is sometimes advantageously added. In some cases of debility, such as are referred to by Dr. Johnson, thinks veratrum may even increase the rapidity of the pulse. If reaction occurs after bleeding, then control with veratrum. Cases frequently die before inflammation is established; thus many claim that it is not an inflammatory disease.

Dr. Taliaferro read an article by Dr. Fordyce Barker, of New York, calling attention to the inaccuracy with which prescriptions, sent to respectable druggists, are frequently compounded. When expensive medicines are ordered, cheaper articles are substituted, the quantity reduced, or that particular ingredient is entirely omitted. Dr. Barker asserts that he has detected the druggist substituting the subnitrate for the subcarbonate of bismuth, and the sulphate of magnesia for the sulphite. Other examples are adduced to show the determined disregard or carelessness of many druggists considered reliable, or ignorance on the part of the clerks employed by them.

Dr. Taliaferro indorsed the position taken by Dr. Barker, and thought it was a subject which should engage the attention of the profession. He held that a prescription clerk should have no other duties than compounding prescriptions, and should not be subject to interruption while so engaged. He had seen a competent druggist interrupted several times while compounding a simple prescription for him, and had found it necessary to stop him and call his attention to errors he was committing. He will prepare no more prescriptions for him.

Dr. J. T. Johnson.—Knowing the carelessness of druggists generally, he always has misgivings when writing an important prescription.

*Extract from Minutes, January* 27, 1873—*Dr. J. P. Logan, President, in the Chair.*

Dr. John G. Westmoreland reported the following case: The subject, an adult male, indulged habitually in the use of intoxicating drinks. When first seen, the stomach refused to retain any alcoholic liquors. He had taken a narcotic, but no alcohol for several hours. He was conscious, and would converse intelligently, when suddenly he would scream out, become insensible and raving. He would occasionally talk to some absent party, and attempt to strike an imaginary person. Was he insane. I believe that he was affected with insanity. I know the law makes a distinction in cases of insanity, but think there is no difference in point of fact. An hallucination is imagining an object is seen where it does not exist. A delusion is a false conception of the mind. Either of these constitute insanity. What is the best treatment in this case? Shall the alcohol be taken away suddenly and entirely, or gradually?

Dr. Taliaferro considers the practice of giving stimulants in mania potu an erroneous one. May have this condition, from excessive use, continued until their sustaining effect fails to be produced. His own treatment consists in the use of bromide of potassium, nux vomica, quinine, capsicum, and this class of remedies, with an eye to complications, as deranged secretions, gastritis, etc. These should be corrected.

Dr. Westmoreland in this case gave twenty grains of chloral; quiet and sleep were produced in twenty minutes.

Dr. J. T. Johnson remarked that he had treated several cases with one-fourth or one-fifth of a grain of morphia hypodermically, and thirty or thirty-five grains of the hydrate of chloral per orem, with the happiest results. He also advised a supporting diet, as patients have frequently eaten nothing for days.

Dr. Connally has seen the condition referred to by Dr. Westmoreland caused by the discontinuance of alcoholic drinks. Uses morphia and chloral combined. Thinks the effect is better than either of these singly. Recalled one case in which two or two and a half grains of morphia had been given hypodermically in a day without satisfactory results; added chloral, and a sleep of six hours' duration followed.

Dr. Taliaferro.—Can understand how the action of either would be augmented by the presence of the other. They both excite the brain, consequently, he thinks bromide of potassium should be combined with them to counteract this tendency to congestion.

Dr. Jno. G. Westmoreland thinks chloral is tranquilizing and quieting. Does the effect seem to warrant the conclusion that it is an excitant of the brain?

Dr. Taliaferro replied that it had been proved in regard to morphine by actual experiment. It is held by some that chloral is converted in the blood into chloroform. We know that chloroform acts in this way. Chloral taken for several consecutive days causes congestion of the conjunctiva, and an uncomfortable feeling of fullness about the head.

Dr. Westmoreland.—Chloroform is a cerebral stimulant; chloral is not. The latter produces sleep; the other occasions vivacity. Chloroform inhaled does produce sleep, but taken into the stomach, it does not. The effect from the two modes is opposite. Never heard of a person being narcotized by a tea-spoonful of chloroform.

Dr. Taliaferro considered it unaccountable that a remedy would produce one effect when taken into the lungs, and another taken into the stomach. They are in both instances absorbed by the blood, and can not produce opposite results.

Dr. J. T. Johnson remarked that always, according to his observation, chloroform by the stomach produces sleep. He remembers no death from chloroform thus administered. In one case, reported to the Academy by him, in which six drachms were taken into the stomach at once, sleep, similar to that from chloral, was produced. The pulse was a little below the normal standard. It causes anæsthesia when taken by the stomach in proper doses—though it irritates the mucous membrane of the stomach.

Dr. Baird inquired the effect of chloral in extensive disease of the lungs, and stated that he had recently given twenty grains to a patient advanced in pulmonary tuberculosis. Several grains of morphine had been previously given without avail. The chloral produced an immediate and profound sleep, but at the same time, for two hours, an alarming depression—the respiration and pulse becoming slow, labored, and intermittent.

Dr. Rauschenberg gives it in five grain doses to an adult, combined with a small portion of morphine, for irritable cough in subacute bronchitis, with the most gratifying results.

Dr. Logan had used it in one case of pneumonia. He was satisfied that the unpleasant symptoms were aggravated by it. He generally prescribed it in ten grain doses, repeated.

Dr. John Stainback Wilson thought the conflicting views of the gentlemen in reference to the action of chloroform were not irreconcilable. In small doses, and the primary effect of large doses, tend to produce excitement; but the full effect of a large dose is that of reduction. It excites when administered by the stomach because only a few drops are given; but when frequently inhaled, three or four ounces are given, and a sedative effect is produced. Like many other remedies, the effect is regulated by the amount taken, and rapidity of introduction.

